# Association between Anti-Ganglionic Nicotinic Acetylcholine Receptor (gAChR) Antibodies and *HLA-DRB1* Alleles in the Japanese Population

**DOI:** 10.1371/journal.pone.0146048

**Published:** 2016-01-25

**Authors:** Yasuhiro Maeda, Kiyoshi Migita, Osamu Higuchi, Akihiro Mukaino, Hiroshi Furukawa, Atsumasa Komori, Minoru Nakamura, Satoru Hashimoto, Shinya Nagaoka, Seigo Abiru, Hiroshi Yatsuhashi, Hidenori Matsuo, Atsushi Kawakami, Michio Yasunami, Shunya Nakane

**Affiliations:** 1 Department of Clinical Research, Nagasaki Kawatana Medical Center, Kawatana, Nagasaki, Japan; 2 Department of Neuroimmunology, Nagasaki University Graduate School of Biomedical Sciences, Nagasaki, Japan; 3 Clinical Research Center, NHO Nagasaki Medical Center, Omura, Nagasaki, Japan; 4 Department of Molecular and Genetic Epidemiology, University of Tsukuba, Tsukuba, Ibaraki, Japan; 5 Department of Hepatology, Nagasaki University Graduate School of Biomedical Sciences, Nagasaki, Nagasaki, Japan; 6 First Department of Internal Medicine, Nagasaki University Graduate School of Biomedical Sciences, Nagasaki, Nagasaki, Japan; 7 Department of Clinical Medicine, Institute of Tropical Medicine, Nagasaki University, Nagasaki, Nagasaki, Japan; 8 Department of Neurology, Graduate School of Medical Sciences, Kumamoto University, Kumamoto, Kumamoto, Japan; Kaohsiung Chang Gung Memorial Hospital, TAIWAN

## Abstract

**Background/Aims:**

Anti-ganglionic nicotinic acetylcholine receptor (gAChR) antibodies are observed in autoimmune diseases, as well as in patients with autoimmune autonomic ganglionopathy. However, the genetic background of anti-gAChR antibodies is unclear. Here, we investigated *HLA* alleles in autoimmune hepatitis (AIH) patients with or without anti-gAChR antibodies.

**Methodology/Principal Findings:**

Genomic DNA from 260 patients with type-1 autoimmune hepatitis (AIH) were genotyped for *HLA-A*, *B*, *DRB1*, and *DQB1* loci. Anti-gAChR antibodies in the sera form AIH patients were measured using the luciferase immunoprecipitation system, and examined allelic association in patients with or without anti-gAChR antibodies.

**Methodology/ Methods:**

We detected anti-α3 or -β4 gAChR antibodies in 11.5% (30/260) of patients with AIH. Among AIH patients there was no significant association between HLA-A, B DQB1 alleles and the positivity for anti-gAChR antibodies. Whereas the HLA-DRB1*0403 allele showed a significantly increased frequency in AIH patients with anti-gAChR antibodies compared with those without anti-gAChR antibodies.

**Conclusions/Significance:**

The frequency of the HLA-DRB1*0403 allele differed among Japanese patients with AIH according to the presence or absence of anti-gAChR antibodies. Our findings suggest that particular HLA class II molecules might control the development of anti-gAChR antibodies in the autoimmune response to gAChR.

## Introduction

Autoimmune autonomic ganglionopathy (AAG) is an acquired channelopathy that is characterized by pandysautonomia [1], in which autoantibodies to ganglionic nicotinic acetylcholine receptors (gAChR) may play a central role [2]. We established the luciferase immunoprecipitation system (LIPS) assay as a new tool to detect anti-gAChR antibodies and demonstrated these auto-antibodies in AAG [3]. Furthermore, using this established anti-gAchR antibody assay system, we reported that a significant proportion of patients with autoimmune diseases were positive for anti-gAChR antibodies [3]. Anti-gAChR antibodies in the patient’ serum recognize (alpha and beta subunits) of the ganglionic acetylcholine receptor [4]. The major histocompatibility complex (MHC) genes are the best documented genetic risk factors for the development of autoimmune diseases and could be involved in autoantibody production [5]. This is because of their expression on antigen-presenting cells, which could favor presentation of autoantigen epitopes to T cells, leading to specific help in autoantibody production [6]. For example, an increased frequency of conserved *HLA-DRB1* domain “shared epitopes” has been demonstrated in rheumatoid arthritis patients with anti-citrullinated peptide antibodies [7]. Similarly, a genetic predisposition to autoantibody production in autoimmune hepatitis (AIH) has been shown to be associated with *HLA* genes [8]. We hypothesized that antibodies against gAChR exist in a subset of AIH patients with genetic susceptibility factors, including *HLA*. We genotyped the *HLA-A*, *B*, *DRB1*, and *DQB1* genes in 260 AIH patients with or without anti-gAChR antibodies and evaluated the association between *HLA* genotype and the production of anti-gAChR antibodies.

## Materials and Methods

### Study population

Consecutive type-1 AIH patients were initially enrolled in the register of the Japanese National Hospital Organization (NHO) liver-network study, contributed to medical facilities in Japan, and prospectively followed since 2009 as a multicenter cohort population [9] All patients satisfied the 1999 revised criteria of International Autoimmune Hepatitis Group (IAIHG) diagnosis of type-1 AIH [10]. Patients were excluded from the study if there was histological evidence of cholangitis or non-alcoholic steatohepatitis. In addition, patients who were positive for hepatitis B virus (HBV)-surface antigen (HBsAg) or hepatitis C virus (HCV)-RNA were excluded. Patients with other causes of liver disease, such as excess alcohol or drug use, were excluded based on reviews of their appropriate history and investigations. The control groups included in this study consisted of 73 healthy controls (HC; mean age, 38.3 ± 11.1 years old, 31 males and 42 females) and 34 subjects with other neurological diseases with any autonomic symptoms (OND; Mean age, 56.3 ± 20.4 years old, 19 males and 15 females). The control group for *HLA-DRB1* genotyping consisted of 120 gender-matched Japanese healthy subjects (6 men and 114 women). The mean ±SD age was 46.0±14.3 years. All of subjects gave their written, informed consent to participate in the present study. The study was approved by the Ethics Committee of National Hospital Organization (NHO) central IRB (H26-2111007).

### Variables at study entry

Demographic and other characteristics of the 230 retained patients were recorded in a database at the initial assessment. Data included sex, age at diagnosis, time of onset of symptoms or other evidence of liver disease, markers of infection with hepatitis viruses HBV and HCV, alcohol intake, coexisting autoimmune diseases, serum levels of ALT, AST, alkaline phosphatase and bilirubin, platelet count and prothrombin time. Anti-nuclear antibodies (ANA) and anti-smooth muscle antibodies (ASMA) were measured by indirect immunofluorescence on HEp-2 cells and cut-off titers for positivity were 1:40. Liver tissue from percutaneous biopsy performed at the referring facility was available for the majority of patients at the time of entry (223/260, 85.8%), but for only a few at the subsequent follow-up examination (8/260, 3.1%).

### *HLA-A*, *B*, -*DRB1* and *-DQB1* genotyping

DNA was extracted from the blood sample and subjected to *HLA-A, B, -DRB1* and *-DQB1*genotype determination by WAKFlow HLA typing kit (Wakunaga Pharmaceutical, Osaka, Japan) based on the reverse sequence-specific oligonucleotide probes method coupled with xMAP technology designed for use with the Luminex system (Luminex Japan, Tokyo, Japan).

### LIPS assay for the detection of autoantibodies to gAChR[3]

Serum antibodies to gAChR were detected by the LIPS assay, as described elsewhere [3]. To generate luciferase reporters for the gAChR α3 and β4 subunits (termed gAChRα3-GL and gAChRβ4-GL, respectively) of the human gAChR, full-length human AChR α3 (P32297, Promega Corporation, Madison, WI, USA) or β4 (P30296, Promega Corporation) was fused to a Gaussia luciferase (GL) mutant (GL^8990^). Human embryonic kidney (HEK) 293F cells (Life Technologies Corportion, Graind Island, NY, USA) were then transfected with the expression plasmid encoding either gAChRα3-GL or gAChRβ4-GL with FuGENE6 (Promega Corporation). Two days later, the transfected cells were solubilized with a Tris-based saline containing 1% Triton^™^ X-100. To detect α3 or β4 gAChR antibodies, 100 μL of the soluble fraction, containing gAChR α3-GL or gAChR β4-GL, was incubated with 15 μL of human serum for 1 hour at 4°C. Subsequently, the fraction was mixed with 15 μL of protein G-sepharose (GE Healthcare, Little Chalfont, Buckinghamshire, UK), 600 μL phosphate-buffered saline (PBS) with 3% bovine serum albumin and 0.05% Tween^®^ 20, and incubated for several hours at 4°C. Following centrifugation and two washes of PBS containing 0.05% Tween^®^ 20, bioluminescence activities of the luciferase reporters in protein G-sepharose were measured with a BioLux^™^ GL assay kit (New England Biolabs, Ipswich, MA, USA) and a Lumat LB 9507 luminometer (BERTHOLD TECHNOLOGIES GmbH & Co. KG, Bad Wildbad, Germany); luminometer output was measured in relative luminescence units (RLU). In order to confirm the accuracy of the LIPS assay for gAChR antibodies, we used commercially available antibodies to human gAChR α3 and β4 (H-100 and S-15; Santa Cruz Biotechnology, Inc., Dallas, TX, USA) as positive controls. Based on anti-gAChRα3 and β4 antibody data from the 73 HCs, cut-off values were calculated as the mean plus 3 standard deviations (SD) from the mean. In this study, antibody levels were expressed as an antibody index (A.I.) that was calculated as follows:

A.I=[measurementvalueofthesampleserum(RLU)]/[thecut-offvalue(RLU)]

The normal value that was established in this study from healthy individuals was <1.0 A.I.

To evaluate the diagnostic accuracy of this assay, we verified the cut-off points for all data collected in the previous study. Cut-off points calculated sensitivity and specificity, and ROC curves were obtained. According to the ROC curves, we confirmed the most discriminative cut-off points. At these points, the sensitivity, specificity, and positive and negative predictive values (PPV and NPV) were calculated. The AUC was 0.849 (95% confidence interval [CI]: 0.786–0.911) for the LIPS assay of the anti-gAChRα3 antibody. With the anti-gAChRα3 antibody cut-off point of 1.0, the sensitivity and specificity were 46.9% (95% CI: 33.7–60.6%) and 99.2% (95% CI: 94.8–100.0%), respectively, and the PPV and NPV were 95.8% and 81.8%, respectively. The AUC was 0.720 (95% CI: 0.632–0.807) for the LIPS assay of the anti-gAChRβ4 antibody. With the anti-gAChRβ4 antibody cut-off point of 1.0, the sensitivity and specificity were 14.3% (95% CI: 6.9–27.1%) and 100.0% (95% CI: 96.2–100.0%), respectively. The PPV and NPV were 100.0% and 74.4%, respectively. This sensitivity and specificity anti-gAChRα3 antibody in our assay system is similar to the previous reports [11].

### Statistics

The distribution of HLA types was compared between patients with or without anti-gAChR antibodies. The risk/protective effects of carrier status of HLA alleles were evaluated by odds ratio (OR) obtained by the comparison of frequencies between patients with or without anti-gAChR antibodies. Comparisons between the 2 groups for phenotype frequencies of each HLA allele were made, and each p value was corrected (Pc), multiplying by the number of tested alleles at each considered locus. *p* values were regarded as significant when they were less than 0.05. Continuous variables were compared using Mann-Whitney tests. All the statistical analyses were performed using the Statistical Analysis System (SAS) and SPSS version 18 software (SPSS, Chicago, IL, USA).

## Results

### Demographic data

Table 1 shows the demographic data for the enrolled type-1 AIH patients. The age at diagnosis ranged from 15 to 88 years (mean, 60.2 ± 12.7 years), which is greater than that in earlier studies on Caucasian patients, and females predominated. In 45 (17.3%) patients, there was concurrent symptomatic autoimmune disease, notably, Hashimoto's disease 18; Sjögren's syndrome 13; Rheumatoid arthritis 13; Basedow disease 2; Primary biliary cirrhosis 1; Systemic lupus erythematosus 1; Multiple sclerosis 1; CREST syndrome 1; Polymyalgia rheumatica 1; Scleroderma 1; Mixed Connective Tissue Disease 1; Idiopathic Thrombocytopenic Purpura 1. Regarding tests for autoantibodies, data for SMA were lacking in 2 and for ANA in 1. Of those tested, 232 (89.6%) gave positive tests (titer > 1:40) for ANA and 36 (40.9%) for SMA. Ninety-eight patients (37.7%) had been treated with prednisolone and 51 (9.6%) with ursodeoxycholic acid alone.

**Table 1:** Baseline Characteristics of 260 Japanese AIH Type 1 Patients.

	n = 260
Gender (male/female)	29/231
Age at presentaion (years)	60.2±12.7
Other autoimmune diseases	45(17.3%)
Baseline Laboratory Values	
AST (IU/L)	450.9±452.9
ALT (IU/L)	500.5±500.1
ALP (IU/L)	463.5±199.2
Total Bilirubin (mg/dl)	3.61±4.89
Albumin (g/dl)	3.83±0.57
IgG (mg/dl)	2431.7±889.7
Platelets (10^4^/μl)	18.8±7.0
ANA + (≧1:40)	232/259(89.6%)
ASMA + (≧1:40)	104/258(40.3%)
Cirrhosis at presentation	40(15.4%)
Received treatment	
Steroid alone	98(37.7%)
Steroid + UDCA	94(36.2%)
Steroid + Aza	9(3.5%)
UDCA alone	51(19.6%)

AIH, autoimmune hepatitis; AST, aspartate aminotransferase; ALT, alanine aminotransferase; ALP, alkaline phosphate; IgG, immunoglobulin G; ANA, anti-nuclear antibody; ASMA, anti-smooth muscle antibody; UDCA, ursodeoxy cholic acid; Aza, azathioprine. Data are expressed as number (percentage) or mean ±standard deviations.

### Clinical characteristics of AIH patients with gAChR antibodies

**Fig 1:**
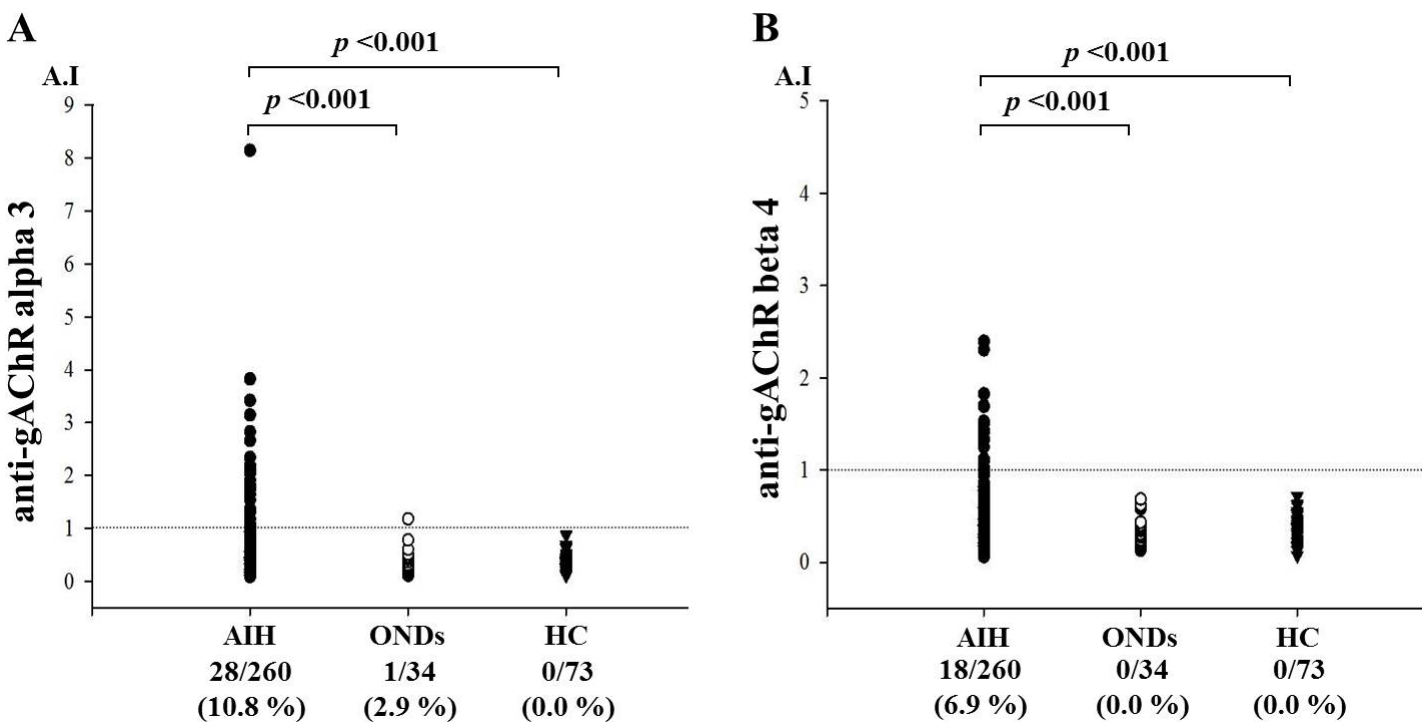
**A) LIPS for gAChR in the sera from patients with autoimmune hepatitis (AIH) and controls.** We tested the sera from patients with AIH, disease controls (ODC), and healthy controls (HC). a) Anti-gAChRα3 antibodies were detected in 28 samples. The mean anti-gAChRα3 antibody level in the HC was 0.305 antibody index (A.I.), which was significantly lower than in the AIH samples with a mean level of 0.609 A.I. (p < 0.001). **B) Anti-gAChRβ4 antibodies were also detected in patient with AIH.** The mean anti-gAChRβ4 antibody levels in the HC was 0.367 A.I., which was significantly lower than the mean levels of 0.506 A.I. in the AIH samples (p < 0.001).

**Fig 2:**
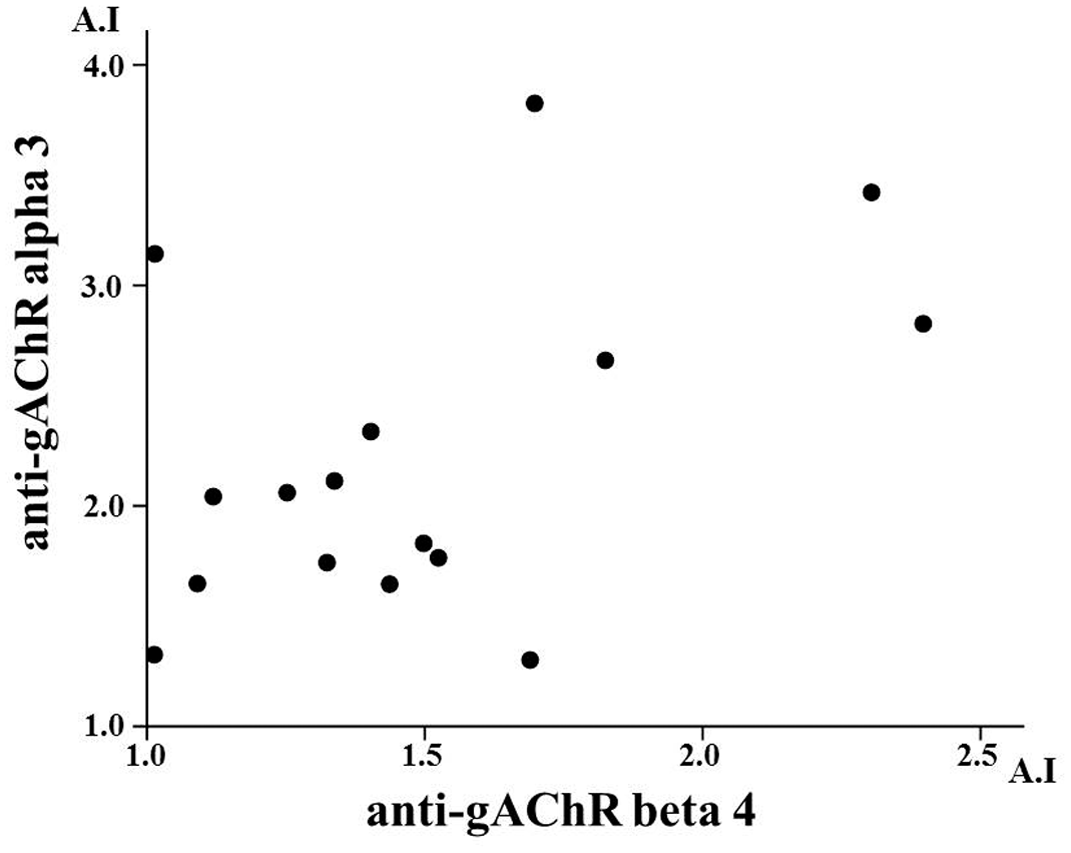
Relationship between anti-gAChR α3 and anti- gAChR β4 antibody levels. Calculation of the correlation between anti-gAChR α3 and anti-gAChR β4 antibody levels in AIH patients with both autoantibodies (n = 17). Correlation was calculated using Spearman’s Rank Correlation test. There is no correlation at all (r = -0.04, *p* = 0.58).

Of the 260 patients with AIH screened, anti-gAChR α3 antibodies were detected in 28 (10.8%) patients and anti-gAChR β4 antibodies were detected in 18 (6.9%) patients. Mean anti-gAChRα3 antibodies in HCs and ONDs were 0.305 A.I. and 0.336 A.I., respectively. These levels were significantly lower than anti-gAChRα3 antibodies in AIH samples, with a mean level of 0.609 A.I. (*p* < 0.001, Fig 1A). Mean anti-gAChRβ4 levels in HCs were 0.367 A.I. and in ONDs was 0.302 A.I., which were significantly lower than that in AIH samples (0.506 A.I., *p* = 0.001, Fig 1B). We investigated the correlation between gAChR α3 and β4 antibodies levels in patients with both antibodies (n = 17). As shown in Fig 2, there was no significant correlation between gAChR α3 and β4 antibodies levels. Also antibodies against particular gAChR single submit have been demonstrated [12]. Therefore, the cross-reactivity of gAChR antibodies against α3 and β4 subunits seems to be unlikely. Table 2 shows the clinical characteristics of AIH patients with or without gAChR antibodies. The gAChR antibody-positive (ab^+^) patients had significantly lower serum albumin levels at the time of registering, as well as lower platelet counts compared with those of the gAChR antibody-negative (ab^–^) patients (Tables 2 and 3).

**Table 2:** Baseline characteristics of AIH patients with or without anti-gAChR Ab (alpha 3).

	anti-gAChR alpha 3		
	positive (n = 28)	negative (n = 232)	*p*
Female	22(78.6%)	209(90.1%)	0.073
Age at presentaion (years)	61.4±12.9	60.0±12.8	0.629
Other autoimmune diseases	5(17.9%)	40(17.2%)	0.554
Baseline Laboratory Values			
AST (IU/L)	370.8±320.8	460.3±465.5	0.729
ALT (IU/L)	414.6±405.8	510.5±509.8	0.580
ALP (IU/L)	439.3±134.8	466.4±205.5	0.751
Total Bilirubin (mg/dl)	2.70±3.36	3.71±5.04	0.865
Albumin (g/dl)	3.62±0.57	3.85±0.56	0.045
IgG (mg/dl)	2644.4±916.2	2406.9±885.4	0.236
Platelets (10^4^/μl)	15.7±7.5	19.1±6.9	0.005
ANA + (≧1:40)	27(96.4%)	205(88.7%)	0.179
ASMA + (≧1:40)	13(46.4%)	91(39.6%)	0.485
Cirrhosis at presentation	5(17.9%)	35(15.1%)	0.438
Received treatment			
Steroid alone	10(35.7%)	88(37.9%)	0.819
Steroid + UDCA	8(28.6%)	86(37.1%)	0.377
Steroid + Aza	1(3.6%)	8(3.4%)	0.647
UDCA alone	7(25.0%)	44(19.0%)	0.447

AIH, autoimmune hepatitis; AST, aspartate aminotransferase; ALT, alanine aminotransferase; ALP, alkaline phosphate; IgG, immunoglobulin G; ANA, anti-nuclear antibody; ASMA, anti-smooth muscle antibody; UDCA, ursodeoxy cholic acid; Aza, azathioprine. Data are expressed as number (percentage) or mean ±standard deviations.

**Table 3:** Baseline characteristics of AIH patients with or without anti-gAChR Ab (beta 4).

	anti-gAChR beta 4		
	positive (n = 18)	negative (n = 242)	*p*
Female	15(83.3%)	216(89.3%)	0.324
Age at presentaion (years)	62.2±14.3	60.0±12.6	0.448
Other autoimmune diseases	3(16.7%)	42(17.4%)	0.620
Baseline Laboratory Values			
AST (IU/L)	357.7±361.3	457.5±458.6	0.439
ALT (IU/L)	441.3±515.3	504.7±499.9	0.392
ALP (IU/L)	452.5±168.8	464.3±201.5	0.974
Total Bilirubin (mg/dl)	3.04±3.93	3.65±4.96	0.864
Albumin (g/dl)	3.51±0.55	3.85±0.56	0.011
IgG (mg/dl)	2451.5±805.2	2430.3±896.8	0.852
Platelets (10^4^/μl)	15.3±8.9	19.0±6.8	0.011
ANA + (≧1:40)	18(100%)	214(88.8%)	0.128
ASMA + (≧1:40)	8(44.4%)	96(40.0%)	0.711
Cirrhosis at presentation	5(27.8%)	35(14.5%)	0.123
Received treatment			
Steroid alone	8(44.4%)	90(37.2%)	0.540
Steroid + UDCA	2(11.1%)	92(38.0%)	0.022
Steroid + Aza	1(5.6%)	8(3.3%)	0.481
UDCA alone	5(27.8%)	46(19.0%)	0.264

AIH, autoimmune hepatitis; AST, aspartate aminotransferase; ALT, alanine aminotransferase; ALP, alkaline phosphate; IgG, immunoglobulin G; ANA, anti-nuclear antibody; ASMA, anti-smooth muscle antibody; UDCA, ursodeoxy cholic acid; Aza, azathioprine. Data are expressed as number (percentage) or mean ±standard deviations.

### *HLA-A*, *B* alleles among AIH patients with anti-gAChR ab

The results of *HLA-A* and *-B* loci typing among AIH patients with anti-gAChR antibodies are summarized in S1–S3 Tables. The frequencies of *HLA-A* and *-B* alleles were not significantly different between gAChR ab^+^ and ab^−^AIH patients.

### Frequencies of *HLA-DRB1* and *DQB1* alleles

**Fig 3:**
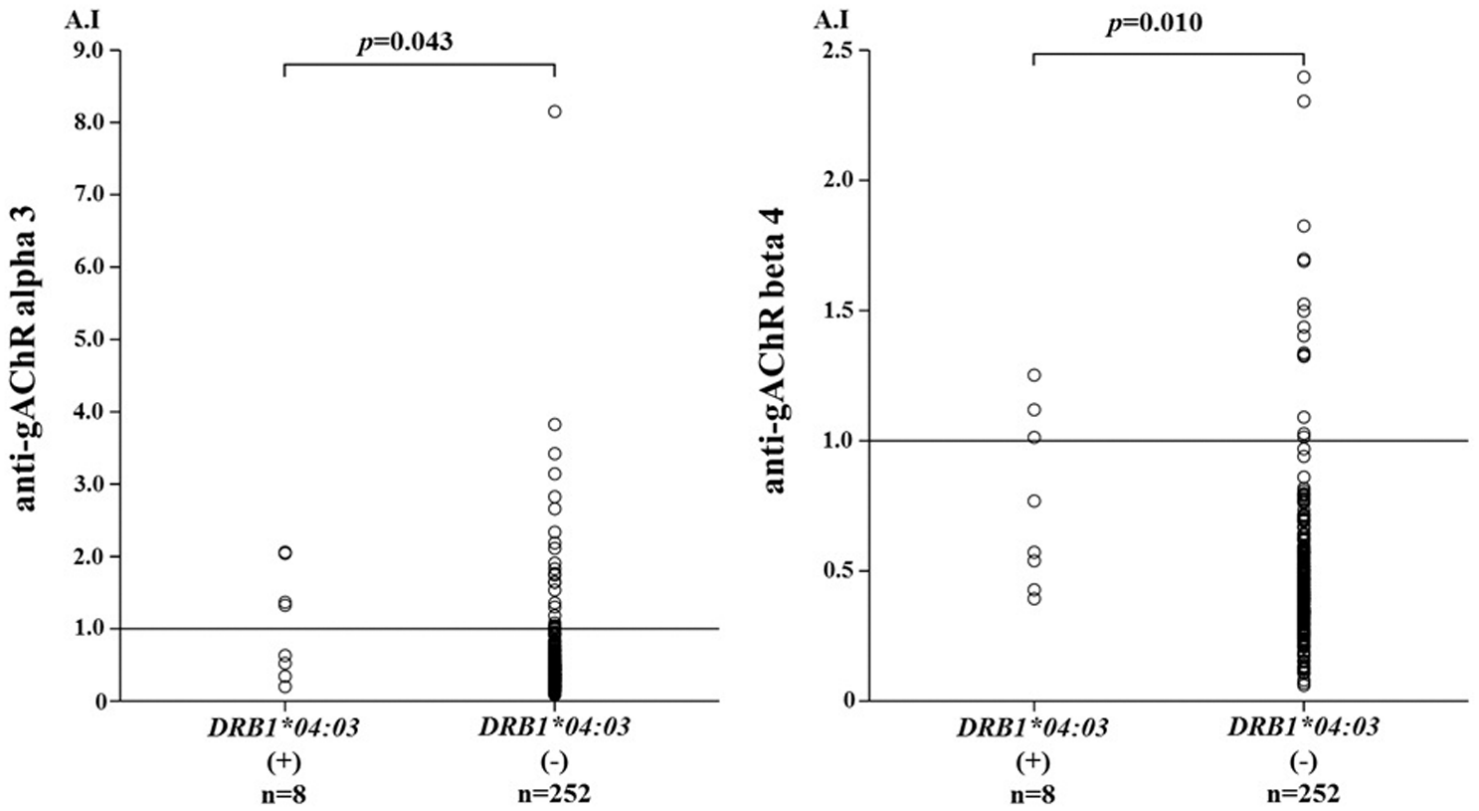
LIPS for gAChR in the sera from AIH patients with or without HLA-DRB1 *0403 allele. We tested the sera from AIH patients with or without HLA-DRB1 *0403 allele. The mean anti-gAChR α3 antibody level in patient with *0403 allele was 1.06 antibody index (AI), which was significantly higher than in those without *0403 allele with a mean level of 0.59 A.I. (*p* = 0.043). The mean anti-gAChR β4 antibody level in patients with *0403 allele was 0.76A.I, which was significantly higher than in the mean level of 0.50A.I. in those without *0403 allele (*p* = 0.010).

As shown in Table 4, gAChR α3 ab^+^ AIH patients had a higher frequency of the DRB1*0403 allele than did gAChR α3 ab^−^patients. Similarly, gAChR β4 ab^+^ AIH patients had a higher frequency of the DRB1*0403 allele compared with gAChR β4 ab^−^patients (Table 5). The frequencies of the *HLA-DRB1* *0403 allele were significantly increased in AIH patients with anti-gAChR α3 or β4 antibodies (Table 6). We compared the antibody index (AI) of anti-gAChR antibodies between AIH patients with or without *HLA-DRB1* *0403 allele (Fig 3). The levels of AI for anti-gAChR α3 in patients with *0403 allele (1.06±0.74 A.I) were significantly higher compared to those without *0403 allele (0.59±0.71 A.I). Similarly, the levels of AI for anti-gAChR β4 antibodies in patients with *0403 allele (0.76±0.33 A.I) were significantly higher compared to those without *0403 allele (0.50±0.32 A.I). Finally, the *HLA-DRB1* alleles distributions in type 1 AIH were compared with gender-matched Japanese healthy subjects. *HLA-DRB1* *0405 allele, not *DRB1* *0403, had been shown to be a genetic factor in Japanese patients with type-1 AIH [13]. In consistent with the previous report [13], the *HLA-DRB1* *0405 alleles were associated with type I AIH, whereas, no significant difference in the frequency of *DRB1* *0403 was present between AIH patients and healthy subjects (Table 7). Therefore, the increased frequencies of AIH susceptible *HLA-DRB1* allele may not linked to the production of anti-gAChR antibody, and a passible association between *DRB1* *0403 and anti-gAchR Ab was suggested.

**Table 4:** HLA-DRB1 alleles of AIH patients with or without anti-gAChR Ab (alpha 3).

anti-gAChR alpha 3				
*HLA* alleles	anti-gAChR alpha 3			
	positive (n = 28)	negative (n = 232)	OR(95%CI)	*Pc*
*HLA-DRB1*				
DRB1*01:*01*	3(10.7%)	11(4.7%)	2.411(0.630–9.225)	ns(5.020)
DRB1*03:*01*	0	1(0.4%)		ns
DRB1*04:*01*	4(14.3%)	12(5.2%)	3.056(0.913–10.221)	ns(1.567)
DRB1*04:*03*	4(14.3%)	4(1.7%)	9.500(2.232–40.432)	0.007
DRB1*04:*05*	13(46.4%)	142(61.2%)	0.549(0.250–1.208)	ns(3.569)
DRB1*04:*06*	2(7.1%)	6(2.6%)	2.897(0.556–15.102)	ns(5.055)
DRB1*04:*07*	0	2(0.9)		ns
DRB1*04:*10*	0	7(3.0%)		ns
DRB1*07:*01*	0	2(0.9%)		ns
DRB1*08:*02*	2(7.1%)	23(9.9%)	0.699(0.156–3.137)	ns(17.239)
DRB1*08:*03*	6(21.4%)	34(14.7%)	1.588(0.600–4.203)	ns(9.397)
DRB1*09:*01*	9(32.1%)	49(21.1%)	1.769(0.754–4.153)	ns(5.014)
DRB1*10:*01*	0	4(1.7%)		ns
DRB1*11:*01*	1(3.6%)	7(3.0%)	1.190(0.141–10.048)	ns(23.559)
DRB1*12:*01*	2(7.1%)	14(6.0%)	1.198(0.258–5.567)	ns(22.077)
DRB1*12:*02*	0	9(3.9%)		ns
DRB1*13:*01*	0	2(0.9%)		ns
DRB1*13:*02*	3(10.7%)	13(5.6%)	2.022(0.539–7.581)	ns(7.770)
DRB1*14:*01*	0	3(1.3%)		ns
DRB1*14:*03*	0	3(1.3%)		ns
DRB1*14:*05*	0	9(3.9%)		ns
DRB1*14:*06*	0	5(2.2%)		ns
DRB1*14:*07*	1(3.6%)	0		ns
DRB1*14:*54*	0	9(3.9%)		ns
DRB1*15:*01*	2(7.1%)	27(11.6%)	0.584(0.131–2.600)	ns(12.835)
DRB1*15:*02*	3(10.7%)	40(17.2%)	0.576(0.166–2.001)	ns(10.256)
DRB1*16:*02*	1(3.6%)	3(1.3%)	2.827(0.284–28.143)	ns(9.580)

Associations were tested by Fisher’s extract test using 2×2 contingency tables. Odds ratio (and its 95% confidence intervals) for the carrier of *HLA-DRB1* were calculated in two groups with or without anti-gAchR. Corrected *p*-valules (*Pc* values) were calculated by Bonferroni's procedure.

**Table 5:** HLA-DRB1 alleles of AIH patients with or without anti-gAChR Ab (beta 4).

anti-gAChR beta 4				
*HLA* alleles	anti-gAChR beta 4			
	positive (n = 18)	negative (n = 242)	OR(95%CI)	*Pc*
*HLA-DRB1*				
DRB1*01:*01*	1(5.6%)	13(5.4%)	1.036(0.128–8.401)	ns(26.283)
DRB1*03:*01*	0	1(0.4%)		ns
DRB1*04:*01*	2(11.1%)	14(5.8%)	2.036(0.425–9.745)	ns(9.837)
DRB1*04:*03*	3(16.7%)	5(2.1%)	9.480(2.066–43.497)	0.015
DRB1*04:*05*	6(33.3%)	149(61.6%)	0.312(0.113–0.860)	ns(0.499)
DRB1*04:*06*	2(11.1%)	6(2.5%)	4.917(0.918–26.341)	ns(1.101)
DRB1*04:*07*	0	2(0.8%)		ns
DRB1*04:*10*	0	7(2.9%)		ns
DRB1*07:*01*	0	2(0.8%)		ns
DRB1*08:*02*	1(5.6%)	24(9.9%)	0.534(0.068–4.194)	ns(14.709)
DRB1*08:*03*	4(22.2%)	36(14.9%)	1.635(0.509–5.248)	ns(10.925)
DRB1*09:*01*	5(27.8%)	53(21.9%)	1.372(0.468–4.020)	ns(15.211)
DRB1*10:*01*	0	4(1.7%)		ns
DRB1*11:*01*	1(5.6%)	7(2.9%)	1.975(0.229–16.993)	ns(14.254)
DRB1*12:*01*	2(11.1%)	14(5.8%)	2.036(0.425–9.745)	ns(9.837)
DRB1*12:*02*	0	9(3.7%)		ns
DRB1*13:*01*	0	2(0.8%)		ns
DRB1*13:*02*	2(11.1%)	14(5.8%)	2.036(0.425–9.745)	ns(9.837)
DRB1*14:*01*	1(5.6%)	2(0.8%)	7.059(0.609–81.828)	ns(1.887)
DRB1*14:*03*	0	3(1.2%)		ns
DRB1*14:*05*	0	9(3.7%)		ns
DRB1*14:*06*	0	5(2.1%)		ns
DRB1*14:*07*	1(5.6%)	0		ns
DRB1*14:*54*	0	9(3.7%)		ns
DRB1*15:*01*	3(16.7%)	26(10.7%)	1.662(0.451–6.125)	ns(11.913)
DRB1*15:*02*	1(5.6%)	42(17.4%)	0.280(0.036–2.163)	ns(5.227)
DRB1*16:*02*	1(5.6%)	3(1.2%)	4.686(0.462–47.497)	ns(4.082)

Associations were tested by Fisher’s extract test using 2×2 contingency tables. Odds ratio (and its 95% confidence intervals) for the carrier of *HLA-DRB1* were calculated in two groups with or without anti-gAchR. Corrected *p*-valules (*Pc* values) were calculated by Bonferroni's procedure.

**Table 6:** HLA-DRB1 alleles of AIH patients with or without anti-gAChR Ab (beta 4).

*HLA* alleles	anti-gAChR alpha 3 or beta 4			
	positive (n = 30)	negative (n = 230)	OR(95%CI)	*Pc*
*HLA-DRB1*				
DRB1*01:*01*	3(10.0%)	11(4.8%)	2.212(0.581–8.429)	ns(6.311)
DRB1*03:*01*	0	1(0.4%)		ns
DRB1*04:*01*	5(16.7%)	11(4.8%)	3.982(1.280–12.391)	ns(0.293)
DRB1*04:*03*	4(13.3%)	4(1.7%)	8.692(2.051–36.839)	0.015
DRB1*04:*05*	13(43.3%)	142(61.7%)	0.474(0.220–1.023)	ns(1.439)
DRB1*04:*06*	2(6.7%)	6(2.6%)	2.667(0.513–13.855)	ns(6.104)
DRB1*04:*07*	0	2(0.9%)		ns
DRB1*04:*10*	0	7(3.0%)		ns
DRB1*07:*01*	0	2(0.9%)		ns
DRB1*08:*02*	2(6.7%)	23(10.0%)	0.643(0.144–2.875)	ns(15.126)
DRB1*08:*03*	7(23.3%)	33(14.3%)	1.817(0.722–4.572)	ns(5.387)
DRB1*09:*01*	9(30.0%)	49(21.3%)	1.583(0.682–3.675)	ns(7.612)
DRB1*10:*01*	0	4(1.7%)		ns
DRB1*11:*01*	1(3.3%)	7(3.0%)	1.099(0.130–9.250)	ns(25.140)
DRB1*12:*01*	2(6.7%)	14(6.1%)	1.102(0.238–5.105)	ns(24.330)
DRB1*12:*02*	0	9(3.9%)		ns
DRB1*13:*01*	0	2(0.9%)		ns
DRB1*13:*02*	3(10.0%)	13(5.7%)	1.855(0.497–6.926)	ns(9.486)
DRB1*14:*01*	1(3.3%)	2(0.9%)	3.931(0.346–44.713)	ns(6.336)
DRB1*14:*03*	0	3(1.3%)		ns
DRB1*14:*05*	0	9(3.9%)		ns
DRB1*14:*06*	0	5(2.2%)		ns
DRB1*14:*07*	1(3.3%)	0		ns
DRB1*14:*54*	0	9(3.9%)		ns
DRB1*15:*01*	3(10.0%)	26(11.3%)	0.872(0.247–3.076)	ns(22.436)
DRB1*15:*02*	3(10.0%)	40(17.4%)	0.528(0.153–1.825)	ns(8.246)
DRB1*16:*02*	1(3.3%)	3(1.3%)	2.609(0.263–25.919)	ns(10.685)

Associations were tested by Fisher’s extract test using 2×2 contingency tables. Odds ratio (and its 95% confidence intervals) for the carrier of *HLA-DRB1* were calculated in two groups with or without anti-gAchR. Corrected *p*-valules (*Pc* values) were calculated by Bonferroni's procedure.

**Table 7:** HLA-DRB1 carrier status in patients with AIH and Healthy controls.

*HLA* alleles	Healthy control	AIH		
	n = 120	n = 260	OR(95%CI)	*pc*
*HLA-DRB1*				
DRB1*01:*01*	12(10.0%)	14(5.4%)	0.512(0.229–1.144)	ns(2.636)
DRB1*03:*01*	1(0.8%)	1(0.4%)	0.459(0.028–7.408)	ns(14.376)
DRB1*04:*01*	2(1.7%)	16(6.2%)	3.869(0.875–17.103)	ns(1.502)
DRB1*04:*03*	1(0.8%)	8(3.1%)	3.778(0.467–30.551)	ns(4.486)
DRB1*04:*05*	39(32.5%)	155(59.6%)	3.066(1.945–4.834)	*p*<0.00001
DRB1*04:*06*	9(7.5%)	8(3.1%)	0.392(0.147–1.041)	ns(1.418)
DRB1*04:*07*	0	2(0.8%)		ns
DRB1*04:*10*	7(5.8%)	7(2.7%)	0.447(0.153–1.303)	ns(3.068)
DRB1*07:*01*	2(1.7%)	2(0.8%)	0.457(0.064–3.286)	ns(10.155)
DRB1*08:*02*	4(3.3%)	25(9.6%)	3.085(1.049–9.072)	ns(0.865)
DRB1*08:*03*	18(15.0%)	40(15.4%)	1.030(0.563–1.884)	ns(24.916)
DRB1*09:*01*	34(28.3%)	58(22.3%)	0.726(0.444–1.189)	ns(5.466)
DRB1*10:*01*	1(0.8%)	4(1.5%)	1.859(0.206–16.816)	ns(13.373)
DRB1*11:*01*	4(3.3%)	8(3.1%)	0.921(0.272–3.119)	ns(15.050)
DRB1*12:*01*	8(6.7%)	16(6.2%)	0.918(0.382–2.208)	ns(22.910)
DRB1*12:*02*	4(3.3%)	9(3.5%)	1.040(0.314–3.446)	ns(16.393)
DRB1*13:*01*	0	2(0.8%)		ns
DRB1*13:*02*	24(20.0%)	16(6.2%)	0.262(0.134–0.515)	0.001
DRB1*14:*01*	0	3(1.2%)		ns
DRB1*14:*03*	4(3.3%)	3(1.2%)	0.339(0.075–1.537)	ns(3.924)
DRB1*14:*05*	2(1.7%)	9(3.5%)	2.116(0.450–9.945)	ns(7.292)
DRB1*14:*06*	3(2.5%)	5(1.9%)	0.765(0.180–3.253)	ns(13.199)
DRB1*14:*07*	0	1(0.4%)		ns
DRB1*14:*54*	5(4.2%)	9(3.5%)	0.825(0.270–2.516)	ns(12.635)
DRB1*15:*01*	15(12.5%)	29(11.2%)	0.879(0.452–1.708)	ns(18.982)
DRB1*15:*02*	30(25.0%)	43(16.5%)	0.594(0.351–1.007)	ns(1.394)
DRB1*16:*02*	1(0.8%)	4(1.5%)	1.859(0.206–16.816)	ns(15.525)

Associations were tested by Fisher’s extract test using 2×2 contingency tables. Odds ratio (and its 95% confidence intervals) for the carrier of HLA-DRB1 were calculated in two groups AIH and Healthy controls. Corrected *p*-valules (*p*c values) were calculated by Bonferroni's procedure.

The frequency of the *HLA-DQB1* allele was not significantly different between gAChR ab^+^ and gAChR ab^−^AIH patients (Table 8).

**Table 8:** HLA-DQB1 alleles of AIH patients with or without anti-gAChR Ab.

anti-gAChR alpha 3				
*HLA* alleles	anti-gAChR alpha 3			
	positive (n = 28)	negative (n = 232)	OR(95%CI)	*Pc*
*HLA-DQB1*				
DQB1*03:*01*	7(25.9%)	50(21.6%)	1.274(0.510–3.184)	ns(6.640)
DQB1*03:*02*	6(22.2%)	25(10.8%)	2.366(0.872–6.416)	ns(0.912)
DQB1*03:*03*	7(25.9%)	51(22.0%)	1.242(0.497–3.102)	ns(7.060)
DQB1*04:*01*	9(33.3%)	71(30.6%)	1.134(0.486–2.646)	ns(8.485)
DQB1*04:*02*	6(22.2%)	80(34.5%)	0.543(0.211–1.399)	ns(2.205)
DQB1*05:*01*	3(11.1%)	15(6.5%)	1.808(0.488–6.698)	ns(4.059)
DQB1*05:*02*	2(7.4%)	10(4.3%)	1.776(0.368–8.566)	ns(5.156)
DQB1*05:*03*	0	14(6.0%)		ns
DQB1*06:*01*	8(29.6%)	73(31.5%)	0.917(0.384–2.192)	ns(9.302)
DQB1*06:*02*	2(7.4%)	24(10.3%)	0.693(0.155–3.110)	ns(6.938)
DQB1*06:*04*	3(11.1%)	12(5.2%)	2.292(0.604–8.695)	ns(2.323)
anti-gAChR beta 4				
*HLA* alleles	anti-gAChR beta 4			
	positive (n = 18)	negative (n = 242)	OR(95%CI)	*Pc*
*HLA-DQB1*				
DQB1*03:*01*	5(27.8%)	52(21.6%)	1.398(0.477–4.100)	ns(5.942)
DQB1*03:*02*	5(27.8%)	26(10.8%)	3.180(1.049–9.639)	ns(0.354)
DQB1*03:*03*	4(22.2%)	54(22.4%)	0.989(0.313–3.130)	ns(10.841)
DQB1*04:*01*	3(16.7%)	77(32.0%)	0.426(0.120–1.515)	ns(1.934)
DQB1*04:*02*	5(27.8%)	81(33.6%)	0.760(0.262–2.205)	ns(6.735)
DQB1*05:*01*	1(5.6%)	17(7.1%)	0.775(0.097–6.181)	ns(8.904)
DQB1*05:*02*	3(16.7%)	9(3.7%)	5.156(1.262–21.056)	ns(0.130)
DQB1*05:*03*	0	14(5.8%)		ns
DQB1*06:*01*	4(22.2%)	77(32.0%)	0.609(0.194–1.910)	ns(4.295)
DQB1*06:*02*	3(16.7%)	23(9.5%)	1.896(0.510–7.039)	ns(3.652)
DQB1*06:*04*	2(11.1%)	13(5.4%)	2.192(0.455–10.565)	ns(3.482)

Associations were tested by Fisher’s extract test using 2×2 contingency tables. Odds ratio (and its 95% confidence intervals) for the carrier of *HLA-DQB1* were calculated in two groups with or without anti-gAchR. Corrected *p*-valules (*Pc* values) were calculated by Bonferroni's procedure.

## Discussion

Although it is accepted that AAG and other autoimmune diseases can coexist because of a background of autoimmunity, few reports have examined anti-gAChR antibodies in autoimmune diseases [14]. We established the LIPS assay as a new tool to detect autoantibodies against gAChR [3]. Using this assay, we demonstrated that a significant number of AIH patients have this autoantibody. This study comprised 260 Japanese patients with AIH, who were carefully selected according to the diagnostic criteria for type 1 AIH. We performed molecular genetic analysis of *HLA* genes in these patients, and compared the findings against anti-gAChR antibody status. We found that anti-gAChR antibody positivity was associated with the HLA-DRB1*0403 allele. Therefore, it is possible that the *HLA-DRB1* gene controls anti-gACHR antibody production and that this production is modulated by particular *HLA-DRB1* alleles. Type-1 AIH is characterized by the presence of anti-smooth muscle antigen (SMA) directed against smooth muscle actin [15]. Whereas anti-gAChR antibodies specifically recognize neuronal α or β subunits of acetylcholine receptor. Therefore, the cross-reactivity of anti-gAChR antibodies against liver antigens such as smooth muscle antigen seems to be insignificant. Furthermore, the associations between anti-SMA antibodies and HLA-B8 or DR3 alleles were reported [16]. Therefore genetic predisposition of type-1 AIH specific autoantibody (anti-SMA antibody) may not affect the production of anti-gAChR antibodies. Type 2 and type 3 autoimmune hepatitis are rare disorders characterized by antibodies against liver-kidney microsome 1 (LKM1) and soluble liver antigens (SLA) [17,18]. Genetic predispositions including HLA for these autoantibodies (anti-SMA. LKM1 and SLA) were shown to be different [19]. We could not recruit these different subtypes of AIH patients in this study. We focused on the type 1 AIH rather than heterogeneous AIH subtypes with differential genetic predispositions and autoantibody profiles.

*HLA-DRB1* is one of the best known polymorphic loci in the human genome, with more than 1700 alleles [20]. *HLA-DRB1* alleles contribute to the development of autoimmune diseases as well as the production of autoantibodies [21]. For the last two decades, scientists have been investigating the relationship between *HLA-DRB1* alleles and autoantibodies [5]. An important discovery was that shared epitope-encoding *DRB1* alleles have been linked to high levels of anti-citrullinated peptide (CCP) antibody in rheumatoid arthritis [22]. Shared epitope-encoding alleles such as DRB1*04:01 and *04:04 that confer rheumatoid arthritis susceptibility are more common in different ethnic populations [23]. Epitope recognition via restriction by the MHC is required for effective T cell help [24]. Documented associations between anti-CCP antibodies and particular HLA class II alleles are likely to reflect presentation of selected T cell determinants. It has been proposed that several positions of the DRB1 molecule independently play a role in antigen presentation according to the nature of the antigen [25].

At present, there is no mechanistic explanation for the association of particular *HLA* alleles with anti-gAChR antibody responses. Although linkage disequilibrium with non-*HLA* alleles cannot be ruled out, it is possible that different HLA class II alleles share relevant amino acid residues in the hypervariable regions, which are considered important for antigen presentation to T cells [26]. Peptide motifs for MHC class II binding and T cell receptors for antigen recognition are presumed to be crucial for immunodominant self-peptides and activation of autoreactive T cells [27]. Recent studies of the *HLA* class II region suggested several candidate amino acids in the hypervariable regions of the *HLA-DRB1* alleles that are predicted to map to the floor of the peptide-binding groove of class II MHC molecules [28]. They could function by presenting antigenic peptides to lymphocytes to initiate anti-gAChR autoimmune responses. We speculate that anti-gAchR antibody production requires efficient help to B cells, which could be provided by DRB1*0403-restricted T helper cells recognizing gAChR determinants. This explanation may account for the differential *DRB1* allele association with anti-gAChR antibodies, but does not give any clue as to the reason for the necessary presence of anti-gAChR antibodies.

DRB1*0403 and *0406 were positively associated with anti-gAChR antibodies, whereas DRB1*0405 was negatively associated in this study. The DRB1*0403 and DRB1*0406 alleles share a Glu amino acid at position 74 [29], whereas the DRB1*0405 allele has Ala at this position [23]; this might imply a role for Glu at position 74 in the development of anti-gAChR antibodies. We suggest a mechanism by which different HLA class II molecules preferentially affect gAChR-specific T helper responses, thereby controlling diversification of the autoantibody response. This hypothesis could be tested by examining the specificity and HLA restriction of anti-gAChR antibodies in patients with other autoimmune diseases.

This study has limitations. Most notably, we did not examine clinical manifestations characteristic of AAG in our AIH patients. Clinical investigations are necessary to confirm the relationship between gAChR antibody positivity and autonomic symptoms. Additionally, it was difficult to perform a replication study due to the very low prevalence of type-1 autoimmune hepatitis and limited numbers of enrolled patients. In this study, we strictly enrolled type-1 AIH patients according to the international diagnostic criteria to maximize diagnostic specificity. Therefore, we could not reserve sufficient sample size, and the possibility of underpowered study cannot be denied. Additionally, it was difficult to perform a replication study due to the very low prevalence AIH.

In conclusion, we have presented evidence that an autoimmune response to gAChR is influenced by different *HLA-DRB1* alleles in Japanese patients with AIH. We suggest a mechanism by which different HLA class II molecules might preferentially affect the gAChR-specific T cell response, controlling diversification of the autoantibody response. This hypothesis should be further tested by examining the HLA restriction of anti-gAChR antibodies in patients with autoimmune diseases. Proof awaits the development of an immunodominant epitope of anti-gAChR antibodies and antigenic motifs for MHC class II binding.

## Supporting Information

S1 Table HLA-A alleles of AIH patients with or without anti-gAChR Ab. (TIF)

S2 Table HLA-B alleles of AIH patients with or without anti-gAChR Ab (alpha 3). (TIF)

S3 Table HLA-B alleles of AIH patients with or without anti-gAChR Ab (beta 4). (TIF)
