# Supplementary figures and images for: Association between Anti-Ganglionic Nicotinic Acetylcholine Receptor (gAChR) Antibodies and HLA-DRB1 Alleles in the Japanese Population
Source: PLoS One. 2016 Jan 25;11(1):e0146048. doi: 10.1371/journal.pone.0146048 (PMC4726510; doi:10.1371/journal.pone.0146048)

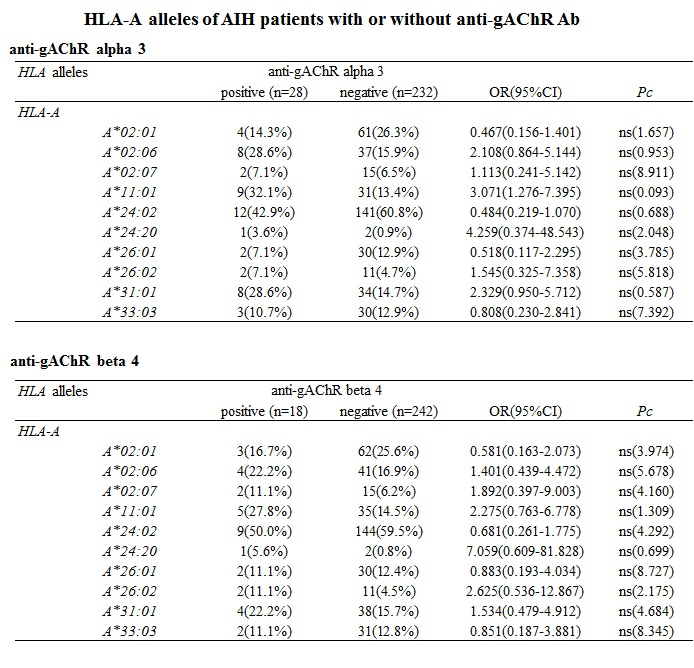

Supplement: S1 Table — (TIF) [file pone.0146048.s001.tif]

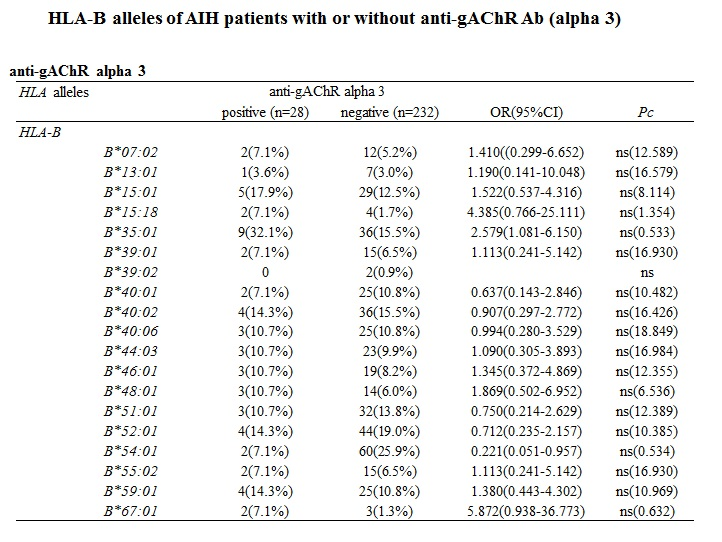

Supplement: S2 Table — (TIF) [file pone.0146048.s002.tif]

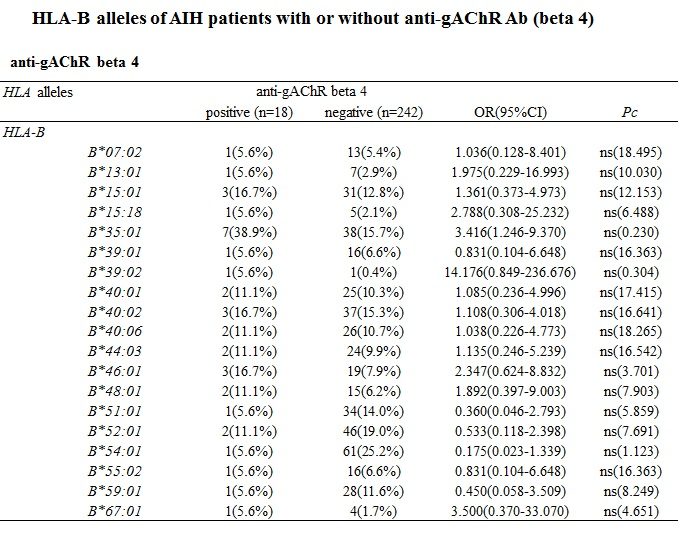

Supplement: S3 Table — (TIF) [file pone.0146048.s003.tif]
